# Accuracy of Smartphone-Mediated Snore Detection in a Simulated Real-World Setting: Algorithm Development and Validation

**DOI:** 10.2196/67861

**Published:** 2025-03-28

**Authors:** Jeffrey Brown, Zachary Mitchell, Yu Albert Jiang, Ryan Archdeacon

**Affiliations:** 1 Bodymatter, Inc, 4343 Von Karman Ave, Suite 150J, Newport Beach, CA, 92660, United States, 1 877-870-0649

**Keywords:** snore detection, snore tracking, machine learning, SleepWatch, Bodymatter, neural net, mobile device, smartphone, smartphone application, mobile health, sleep monitoring, sleep tracking, sleep apnea

## Abstract

**Background:**

High-quality sleep is essential for both physical and mental well-being. Insufficient or poor-quality sleep is linked to numerous health issues, including cardiometabolic diseases, mental health disorders, and increased mortality. Snoring—a prevalent condition—can disrupt sleep and is associated with disease states, including coronary artery disease and obstructive sleep apnea.

**Objective:**

The SleepWatch smartphone app (Bodymatter, Inc) aims to monitor and improve sleep quality and has snore detection capabilities that were built through a machine-learning process trained on over 60,000 acoustic events. This study evaluated the accuracy of the SleepWatch snore detection algorithm in a simulated real-world setting.

**Methods:**

The snore detection algorithm was tested by using 36 simulated snoring audio files derived from 18 participants. Each file simulated a snoring index between 30 and 600 snores per hour. Additionally, 9 files with nonsnoring sounds were tested to evaluate the algorithm’s capacity to avoid false positives. Sensitivity, specificity, and accuracy were calculated for each test, and results were compared by using Bland-Altman plots and Spearman correlation to assess the statistical association between detected and actual snores.

**Results:**

The SleepWatch algorithm showed an average sensitivity of 86.3% (SD 16.6%), an average specificity of 99.5% (SD 10.8%), and an average accuracy of 95.2% (SD 5.6%) across the snoring tests. The positive predictive value and negative predictive value were 98.9% (SD 2.6%) and 93.8% (SD 14.4%) respectively. The algorithm performed exceptionally well in avoiding false positives, with a specificity of 97.1% (SD 3.5%) for nonsnoring files. Inclusive of all snoring and nonsnore tests, the aggregated accuracy for all trials in this bench study was 95.6% (SD 5.3%). The Bland-Altman analysis indicated a mean bias of −29.8 (SD 41.7) snores per hour, and the Spearman correlation analysis revealed a strong positive correlation (*r*_s_=0.974; *P*<.001) between detected and actual snore rates.

**Conclusions:**

The SleepWatch snore detection algorithm demonstrates high accuracy and compares favorably with other snore detection apps. Aside from its broader use in sleep monitoring, SleepWatch demonstrates potential as a tool for identifying individuals at risk for sleep-disordered breathing, including obstructive sleep apnea, on the basis of the snoring index.

## Introduction

The central importance of high-quality sleep in the maintenance of physical and mental health cannot be overstated, as well as the critical role of sleep in avoiding disease states and other physiologic impairments. Insufficient or poor-quality sleep has extensively documented associations with an array of negative consequences that affect both personal and public health. These issues include cardiometabolic disease states, such as hypertension, diabetes, and obesity, as well as increased frequency of heart attacks and strokes [1-3]. Sleep disruption profoundly impacts mental health and emotional well-being, increasing the frequency of depression, anxiety, inattentiveness, learning and memory impairment, and mood disorders [4-6]. From a public health perspective, poor-quality sleep correlates with workplace and vehicular accidents, and health issues arising from sleep impairment impart a massive economic burden to the health care system, as well as an increase in all-cause mortality [2,7-9].

Although the health risks of insufficient or poor-quality sleep are well known among the scientific community and society at large, most individuals struggle to obtain sleep of appropriate quality and quantity. The frequency of insufficient sleep varies by study, but as a conservative estimate, at least one-third of the US adult population reports regularly sleeping less than the recommended 7 hours each night [10-12]. Moreover, there is evidence that sleep problems are worsening over time globally; the percentage of US adults reporting short sleep durations (defined as less than 6 h) has consistently increased over the last several decades [13,14]. The precise cause of this declining trend in sleep health is multifactorial but is influenced in large part by poor sleep hygiene related to the ongoing technological evolution of the modern world [15,16]. These concerns emphasize the need for interventions to address the profound personal and societal issue of sleep impairment.

Among other factors related to poor-quality sleep, a noteworthy and underappreciated cause of sleep impairment is the physiological phenomenon of snoring. Snoring is an auditory process that occurs during sleep as a result of air passing over relaxed tissues of the oropharynx, which produces vibrations and the sounds that are classically associated with snoring. Snoring is extremely common, especially among older adults, male individuals, and individuals with obesity; the reported frequency of habitual snoring among adults ranges from 20% to 60%, depending on risk factors [17-20]. Snoring is often benign but may be a source of personal embarrassment and a cause of relationship distress between sleep partners [19,21]. On a more concerning note, snoring may be associated with serious clinical conditions, including coronary artery disease, obstructive sleep apnea, and depressive disorders [17,22,23].

As with other areas of health optimization, smartphone-based apps have been developed to help address the need for improved sleep quality and sleep duration, and several such apps include snore detection capabilities. Because snoring occurs during sleep, individuals often lack insight into how frequently or how intensely they snore, and obtaining this information through formal polysomnography can be inconvenient, time-consuming, and cost-prohibitive. As such, there is an unmet need for novel, personalized methods of monitoring sleep quality, including evaluation of snoring and sleep-disordered breathing. There are several smartphone-based snoring detection apps in existence, and while these apps are promising in terms of their cost and scalability, most lack rigorous validation, and questions remain with regard to their real-world accuracy for individual users. This paper highlights the results of a bench study that evaluated a proprietary snore detection algorithm in a simulated real-world setting.

## Methods

### Study Design

The smartphone-based, acoustical snore detection algorithm used in the SleepWatch app was developed by the Bodymatter team in-house, using a deep neural net model trained on over 60,000 individually validated, real-world snore and nonsnore sounds. The SleepWatch snore detection capabilities were then tested against an array of snoring and sleep audio files in a controlled acoustic setting. Sleep audio files were derived from volunteers in an anonymized fashion, as outlined in the *Ethical Considerations* subsection. No raw or identifying human participant data were used in this study. Additionally, all training data were derived entirely from audio sources that were separate from the validation and test audio sets used in this bench study and prior trials.

In total, 36 trials were conducted based on test audio files that were 10 minutes in duration and were derived from 18 individual SleepWatch participants (male: n=9; female: n=9). Audio quality fidelity of the test files was ensured during their editing, exporting, and uploading. There were 2 audio files made from each participant, simulating a snoring index of 30, 60, 120, 240, 360, or 600 snores per hour. For each file with a snoring index of 120, 240, 360, or 600 snores per hour, 10 unique snores were repeated at regular intervals. For each file with a snoring index of 30 or 60 snores per hour, 5 unique snores were repeated at regular intervals. The duration of snore events was limited to a maximum of 5 seconds, in accordance with the scoring methodology described further in this paper. The intervening audio between inserted acoustic events was the ambient noise present at baseline during a given participant’s sleep recording. Additionally, 9 trials were conducted based on test audio files that exclusively contained confounding, nonsnoring audio events (including percussive sounds, coughing, speech sounds, movement of bedding, etc). These confounding audio events reflected common ambient noise that was expected during typical sleeping conditions and had the potential to be erroneously detected as a snore. Again, no confounding acoustic event lasted longer than 5 seconds, in accordance with the scoring methodology. Of note, any audio samples that were determined to have corruption at any level of the editing or exporting process and resulted in distorted playback were discarded.

For each trial, a SleepWatch sleep recording session was started on an iPhone 13 smartphone (Apple Inc) that ran the SleepWatch app (v8.2.3.0-pkf33, public release v8.3.0) shortly before simultaneously starting the test audio playback. Test audio files were played from a dedicated JBL 305P MKII (Harman International Industries, Inc) studio speaker in close proximity to the detection smartphone to simulate product implementation in a real-world setting, where users would place their smartphone within 5 feet of their sleeping position. Trials were conducted under acoustically controlled ambient conditions. For each test, audio playback intensity was confirmed via an independent decibel meter and limited to a 125-millisecond (fast response) peak of 65 A-weighted decibels (dB[A]) and a floor of 50 dB(A). Trials were performed for 10 minutes, after which the SleepWatch app performance was recorded.

After each trial, the SleepWatch-detected snore count was compared against the known number of snoring events present in each audio file. Additionally, app performance was assessed by reporting sensitivity (true-positive rate), specificity (true-negative rate), and accuracy (ratio of true events to total events). As a scoring method, each audio file was discretized into 5-second intervals (N=120). An appropriately identified snoring event was considered a true-positive event for a 5-second interval, and conversely, a sound that was inaccurately reported as snoring was considered a false-positive event for the corresponding 5-second interval. False-negative events were missed snores, and true-negative events were 5-second regions of silence or ambient noise that were not reported as snoring. Sensitivity was calculated as “(true positive)/(true positive + false negative),” and specificity was calculated as “(true negative)/(true negative + false positive).” Positive and negative predictive values were also determined, calculated as “(true positive)/(true positive + false positive)” and “(true negative)/(false negative + true negative),” respectively. Accuracy was determined as “(true positive + true negative)/all events” (all events: N=120). In the case of the 9 trials that used confounding nonsnore sounds, only specificity and accuracy could be described due to the absence of positive events, and this was factored into the aggregate accuracy. This scoring methodology is summarized in Table 1.

After the initial analysis, a Bland-Altman plot, which compared the average snoring index (snores/h) and difference in the snoring index between the app and test audio files, was produced as a visual method of analyzing bias associated with the app’s performance. A Spearman correlation was performed between known and detected snoring index values, and the correlation coefficient (*r*_s_) and statistical significance were determined. All statistical analyses were completed by using IBM SPSS Statistics (version 29.0.2.0; IBM Corp).

**Table 1.** Analytical validation reference table showing the schematic and equations^a^^,^^b^^,^^c^^,^^d^^,^^e^ used to report snore detection performance of the SleepWatch app in this study.

		Constructed sleep audio (gold standard)	
		Snoring occurred	Snoring did not occur
Smartphone app			
	Snoring detected	True positive	False positive
	Snoring not detected	False negative	True negative

a Accuracy = (true positive + true negative)/(true positive + true negative + false positive + false negative).

b Sensitivity = true positive/(true positive + false negative).

c Specificity = true negative/(false positive + true negative).

d Positive predictive value = true positive/(true positive + false positive).

e Negative predictive value = true negative/(false negative + true negative).

### Ethical Considerations

A principal aim of this study was to closely reproduce a real-world environment for the validation of Bodymatter software. As such, raw audio files were voluntarily obtained from individuals who were willing to participate in this study. Written consent was obtained for the use of their sleep audio for internal testing purposes. No compensation was provided to participants for their participation in this study. All audio files were anonymized at both the level of audio editing and the level of audio playback to ensure participant privacy. As no direct intervention was performed on study participants and this study posed no risk to participants, this study was granted an exemption from formal institutional review board approval for human subjects research.

## Results

Across the 18 participants in this study, 9 were male and 9 were female. The age distribution skewed toward a younger adult demographic, with 28% (5/18) of participants aged 25 to 34 years, 44% (8/18) aged 35 to 44 years, 22% (4/18) aged 45 to 54 years, and 6% (1/18) aged 55 to 64 years. The average BMI was 31.21 (SD 5.03) kg/m^2^ for male participants and 33.93 (SD 8.07) kg/m^2^ for female participants. With respect to the presence of sleep partners, 56% (10/18) of participants reported sleeping alone, and 44% (8/18) reported having a consistent sleep partner.

The snore detection performance was evaluated for the 36 snoring tests and 9 confounding noise tests. During the development and internal validation of the SleepWatch snore detection algorithm, several trends were observed. The SleepWatch snore detection algorithm has a strong capacity to appropriately designate true-positive events (sensitivity) and an extremely strong capacity to appropriately define true-negative events (specificity); that is, the algorithm almost never reports a snore when one did not occur. Table 2 reports the sensitivity, specificity, and accuracy for both the snoring tests and the confounding audio (nonsnore) tests. The cumulative sensitivity for the snoring tests was 86.3% (SD 16.6%), the cumulative specificity was 99.5% (SD 10.8%), and the cumulative accuracy was 95.2% (SD 5.6%). The positive and negative predictive values were 98.9% (SD 2.6%) and 93.8% (SD 14.4%), respectively.

Consistent with the performance observed for the dedicated snore audio files, the SleepWatch algorithm continued to perform exceptionally well for the dedicated nonsnore audio files, with an overall specificity (ie, the true-negative capacity) of 97.1% (SD 3.5%). The sensitivity, positive predictive value, and negative predictive value cannot be reported, as there were no snoring or positive events in these audio files, but a false-positive rate of 2.9% was observed across all the noise audio files—defined as the ratio of erroneously detected snores to the total number of nonsnoring time intervals. Inclusive of the nonsnore audio files, the aggregated accuracy for all trials in this bench study was 95.6% (SD 5.3%).

**Table 2.** Detection performance of the SleepWatch app for cumulative, snoring, and nonsnore noise audio tests. The results were produced from 36 snoring trials and 9 nonsnoring noise trials.

Test condition	Sensitivity (%), mean (SD)	Specificity (%), mean (SD)	Positive predictive value (%), mean (SD)	Negative predictive value (%), mean (SD)	Accuracy (%), mean (SD)
Snore audio	86.3 (16.6)	99.5 (10.8)	98.9 (2.6)	93.8 (14.4)	95.2 (5.6)
Nonsnore audio	—^a^	97.1 (3.5)	—	—	97.1 (3.5)
Cumulative	86.3 (16.6)	98.0 (9.8)	98.9 (2.6)	93.8 (14.4)	95.6 (5.3)

a Not applicable.

Of note, the detected false-positive events were not evenly distributed across files. Algorithm performance was strong for coughing, percussive sounds, and loud speaking, for which no false-positive events were observed. Intermediate performance was observed for movement sounds, such as sounds resulting from pacing around a room, climbing stairs, or rustling pillows or bedding. Algorithm performance was poor for a specific file that included whispering and sleep talking, for which a false-positive rate of 10.8% was observed.

**Figure 1.**
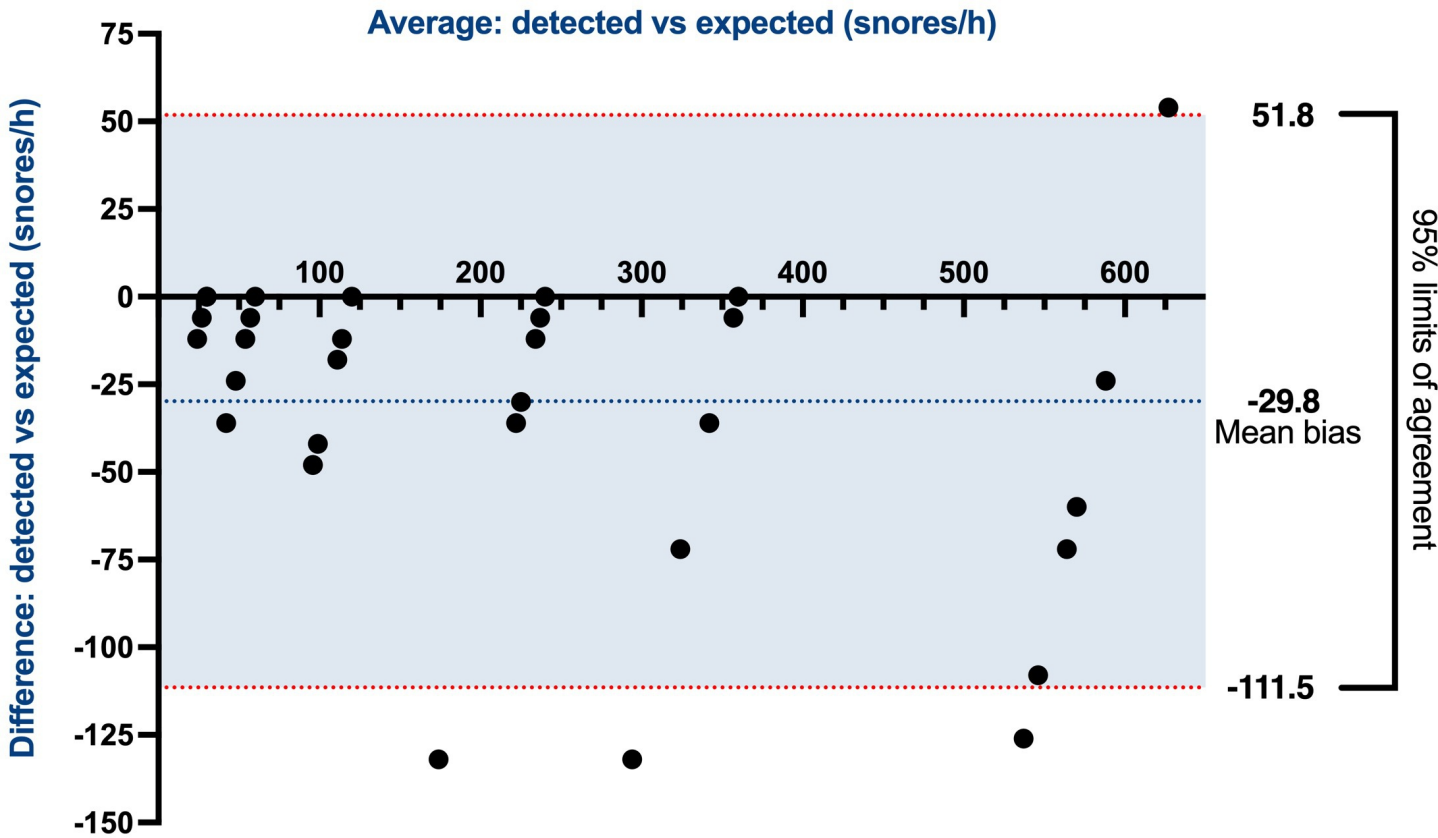
Bland-Altman plot of the difference in and average of the detected versus expected snores/h. The Bland-Altman plot demonstrates a mean bias of –29.8 (95% limits of agreement –111.5 to 51.8) snores/h for the average of the detected versus expected snores/h graphed against the difference in the detected versus expected snores/h. This demonstrates a relatively tight relationship between detected and expected snores.

The results of the 36 snoring tests were compiled and displayed in a Bland-Altman plot (Figure 1). The average of the known versus detected snoring index (snores/h) was plotted on the x-axis, and the difference in the known versus detected snoring index was plotted on the y-axis. As expected, there were roughly 6 vertical data clusters around the x values 30, 60, 120, 240, 360, and 600 snores per hour, corresponding to the planned variations in snoring rate for each audio file. If the average of the known versus detected snores per hour aligned closely with these six x values, then excellent performance was interpreted. In contrast, if the difference in the known versus detected snores per hour was close to 0 on the y-axis across all tests, irrespective of the snoring rate, then ideal performance was also indicated. The mean bias of the SleepWatch app for this study was −29.8 (SD 41.7) snores per hour, indicating an average difference of approximately 30 snores per hour between the detected and expected snores. Most values were contained within the 95% limits of agreement (51.8 and −111.5 snores/h).

**Figure 2.**
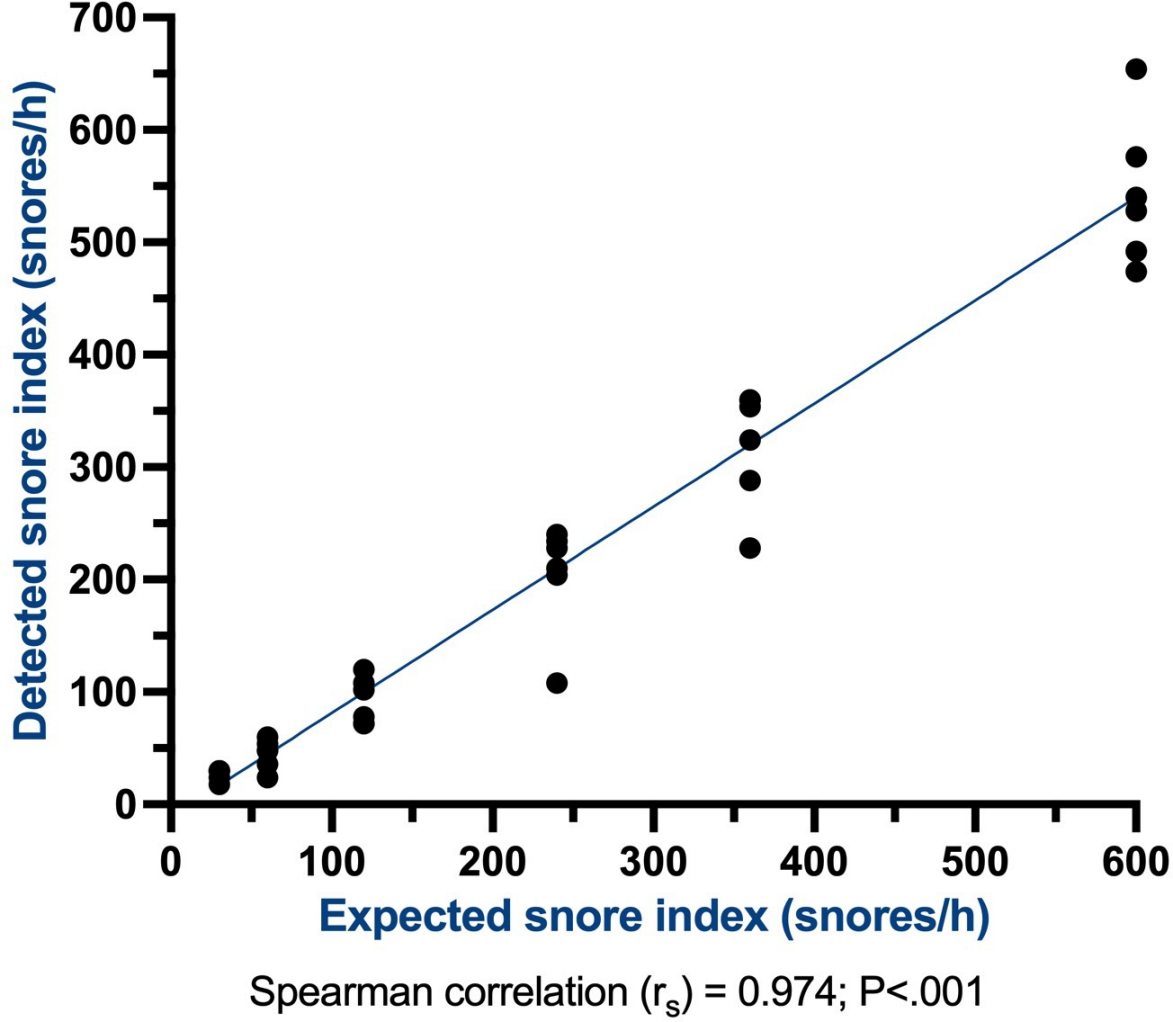
The expected snore index graphed against the snore index detected by the SleepWatch app. This demonstrates a tight grouping and a Spearman correlation coefficient of 0.974, indicating a strong, positive correlation between expected and detected snores.

A Spearman correlation coefficient was calculated to statistically evaluate the association between the detected and known snoring index (snores/h) in each test audio file (Figure 2). The Spearman coefficient (*r*_s_) for this cohort was 0.974, denoting an extremely strong, positive correlation between detected snores and expected snores (*P*<.001). These findings are highly encouraging and demonstrate a robust capacity for identifying true snores in a simulated real-world setting.

## Discussion

### SleepWatch Snore Detection Accuracy Compares Favorably to Similar Snore Detection Modalities

The SleepWatch app provides users with consistent, valuable insight into their sleep quality, and the snore detection feature constitutes an accuracy benchmark for acoustic snore detection software. This study reports the performance of the SleepWatch snore detection algorithm in detail; the algorithm showed an average sensitivity of 86.3% (SD 16.6%), an average specificity of 99.5% (SD 10.8%), and an average accuracy of 95.2% (SD 5.6%). Furthermore, the accuracy of the SleepWatch snore detection feature reflects performance that is superior to that of similar apps, and this feature is solely dependent on existing smartphone hardware; thus, it has powerful implications for improving sleep quality and screening for sleep-disordered breathing on an individualized basis.

There are several snore detection modalities in existence that have published results with respect to the accuracy of snore detection. The accuracy, sensitivity, specificity, positive predictive values, and negative predictive values of such modalities are summarized in Table 3, though these results must be compared with caution due to the differing methodologies between studies. Shin et al [24] integrated and analyzed a series of sleep and noise datasets for 10 individuals, attempting to distinguish verified snoring from ambient noise (playing music, running a fan, talking, etc). They reported a 95.1% overall accuracy in detecting snores and a sensitivity and specificity of 98.6% and 94.6%, respectively, as well as a positive and negative predictive value of 70.4% and 99.8%, respectively. Chiang et al [25] evaluated the sleep records of 11 patients using the Snore Clock app. The authors reported an overall mean snore detection accuracy of 95% and a sensitivity and specificity of 78.2% and 97%, respectively, along with a positive and negative predictive value of 65.3% and 97%, respectively. They also commented that the false-negative rate (type II error) was higher in patients with greater snoring frequencies. In a cohort of 19 patients, Klaus et al [26] compared the performance of the SnoreLab app with that of formal polysomnography, reporting an accuracy of 94.7% and a sensitivity and specificity of 100% and 94.1%, respectively, as well as a positive and negative predictive value of 66.6% and 100%, respectively. These findings leave room for misinterpretation however, as these statistics were observed only in a group of participants that snored for >50% of the time recorded, constituting approximately 10% of the cohort. Inclusive of users who snored for >5% of the time recorded—a far less restrictive threshold comprising 42% of the participants—the accuracy, sensitivity, and specificity were 73.6%, 100%, and 54.5%, respectively, with a positive and negative predictive value of 61.5% and 100%. Klaus et al [26] also noted that the app had a strong tendency to overestimate the ratio of snoring time to quiet sleep time, relative to the comparison polysomnogram.

**Table 3.** Cross-study comparison of our study’s snore detection app and other snore detection modalities reported in available literature. Our study’s modality compares favorably against similar snore detection modalities relying on acoustic input.

Study	Accuracy, %	Sensitivity, %	Specificity, %	Positive predictive value, %	Negative predictive value, %
Our study	95.6	86.3	99.5	98.9	93.8
Shin et al [24]^a^	95.1	98.6	94.6	70.4	99.8
Klaus et al [26]^b^	73.6	100	54.5	61.5	100
Chiang et al [25]^c^	95	78.2	97	65.3	97

a Shin et al [24] only used a smartphone for audio recording; snore detection was processed through a separate modality.

b Statistics from the Klaus et al [26] study are reported for participants with a snoring ratio of >5%, inclusive of the largest subset of participants evaluated.

c Chiang et al [25] selected participants from patients with known sleep apnea who were using an oral device that was intended for reducing snoring.

Collectively, the previously mentioned studies suggest that there is reasonable accuracy for smartphone-based snoring detection software, but there are several limitations with comparing our study to those snore detection studies. Notably, only 2 of those studies used working smartphone apps that recorded and processed snoring. Shin et al [24] only used a smartphone as a recording device and processed sleep audio and snore detection through a separate modality. Chiang et al [25] and Klaus et al [26] used smartphone apps with a methodology that was consistent with our study. However, Chiang et al [25] recruited participants with known sleep-disordered breathing or obstructive sleep apnea who had received a mandibular advancement device that was intended for reducing airway obstruction and snoring, which could potentially introduce a degree of selection bias and thereby limit cross-study comparison. Further, as previously stated, Klaus et al [26] reported results for an arbitrarily defined cohort that was considered to have spent >50% of the sleep session snoring, relative to quiet sleeping. App performance was far worse when the snoring threshold was >5%, with an accuracy of 73.6% in that subcohort. This discrepancy in methodology imparts limitations in the comparison of snore detection statistics between the study by Klaus et al [26] and our study, making cross-study comparison largely irrelevant.

The SleepWatch app by Bodymatter stands out for several reasons. Given that only 2 of the previously referenced studies involved a working smartphone app running snore detection software, many of the processes and protocols in the referenced studies do not seem practical to implement on an individualized, large-scale basis, whether due to concerns regarding privacy or concerns regarding convenience. Furthermore, the SleepWatch snore detection feature far outcompetes the referenced studies’ modalities in positive predictivity and specificity, which, in practical language, denotes that (1) there is an extremely high likelihood that a true snore actually occurred when a snore was identified by the algorithm, and (2) the app has an extremely strong capacity to appropriately discriminate silence and nonsnoring noise events, with the latter representing one of the most significant challenges in snore detection. This reflects an engineering priority of avoiding false-positive reported snores in the development of the Bodymatter SleepWatch algorithm. Finally, this study carries the advantages of reporting performance on a snore-by-snore basis and including ambient noise and confounding sound events in the testing of the snore detection software, thereby evaluating performance in conditions that more closely reflect real-world settings and expected use.

### Screening for Sleep-Disordered Breathing

As previously described, snoring is independently associated with an array of disease states, and snore detection smartphone apps provide individual users with insight into the presence and frequency of their snoring. Loud snoring is a cardinal symptom of obstructive sleep apnea—a disease characterized by airway collapse during sleep, which results in intermittent deoxygenation, apneic events, and arousal at night [27]. Furthermore, obstructive sleep apnea results in daytime sleepiness and an associated increase in accidents, as well as an increased risk of hypertension, coronary artery disease, depression, and death [28,29].

Diagnostic criteria for obstructive sleep apnea center around the apnea-hypopnea index (AHI), which describes the number of episodes where an individual stops breathing due to airway obstruction (apnea) or experiences decreased oxygenation due to inadequate breathing (hypopnea). A diagnosis of moderate-severity sleep apnea is made when >15 apneic or hypopneic events occur in 1 hour (AHI>15) [30]. Previous studies have described a positive correlation between the snoring index and AHI and, thereby, a positive correlation with the diagnosis of obstructive sleep apnea [31,32]. Moreover, it has been estimated that as many as 85% of individuals with obstructive sleep apnea are undiagnosed, highlighting the importance of novel, affordable, and scalable tools for disease screening [33].

These findings suggest that there is utility in exploring the SleepWatch app as a screening tool for stratifying users along a risk spectrum for sleep-disordered breathing, including obstructive sleep apnea. As previously mentioned, the snoring index alone is a useful metric for assessing user risk, aside from formal diagnostic criteria, such as apneic and hypopneic events. Ultimately, there is both significant need and potential for at-home technological innovations to assist with screening for underdiagnosed health conditions, including obstructive sleep apnea, and SleepWatch may have potential uses in a clinical context, aside from providing individual users with insights into their sleep health.

### Limitations

Although the reported results of the SleepWatch snore detection feature are promising, this study must be evaluated with several limitations in mind. As mentioned previously, while false-positive snore events were infrequent, it is difficult to entirely eradicate such events. This is a priority for the future development of the snore detection algorithm. Further, this initial study involved a relatively small cohort, and a study with a larger sample size might more comprehensively evaluate the performance of the snore detection algorithm, due to a greater degree of acoustic heterogeneity. Additionally, there are intrinsic limitations in participant-derived audio collection, as individuals may place their phones at varying distances from the sleeping location, and the ambient acoustic environment naturally differs from person to person. From another perspective however, this acoustic diversity may represent a strength of this study, as it provides evidence that app performance is robust, irrespective of ambient sound conditions.

### Conclusion

The SleepWatch app by Bodymatter has a strong track record of providing users with individualized insights into their sleep hygiene and represents a powerful and scalable modality for improving overall sleep quality. This study evaluated the performance of the deep neural net algorithm powering the SleepWatch snore detection feature. Over a series of trials involving snoring and nonsnoring sounds from 18 participants, a strong, positive correlation (*r*_s_=0.974; *P*<.001) was found between detected and reference snoring indices that ranged from 30 to 600 snores per hour. The snore detection feature exhibited an overall accuracy (95.6%) that was competitive with those of similar modalities, as well as a remarkable ability to discriminate true snores from nonsnores, as reflected by the superior specificity and positive predictive value. This positions SleepWatch as a powerful tool for monitoring sleep quality and snoring. Although financial, geographic, and time constraints may prevent individuals from accessing traditional health and wellness resources, SleepWatch represents an accessible, scalable, and intuitive instrument for monitoring an individual’s snoring over time and potentially identifying individuals at risk for sleep-disordered breathing, thus helping reduce the growing disease burden of impaired sleep.

## Supplementary material

10.2196/67861 Multimedia Appendix 1 Snore detection results broken down by participant, with app performance metrics for each individual.

## Abbreviations

AHI: apnea-hypopnea index
dB(A): A-weighted decibels
