# Supplementary material for: Accuracy of Smartphone-Mediated Snore Detection in a Simulated Real-World Setting: Algorithm Development and Validation
Source: JMIR Form Res. 2025 Mar 28;9:e67861. doi: 10.2196/67861 (PMC11970566; doi:10.2196/67861)
Supplement: Multimedia Appendix 1 [file formative-v9-e67861-s001.pdf]

Participant	True Positive	False Positive	True Negative	False Negative	Sensitivity	Specificity	Accuracy	Positive Predictive Value	Negative Predictive Value
1F	5	0	115	0	100.0%	100.0%	100.0%	100.0%	100.0%
2F	4	0	110	6	40.0%	100.0%	95.0%	100.0%	94.8%
3F	17	0	100	3	85.0%	100.0%	97.5%	100.0%	97.1%
4F	35	0	80	5	87.5%	100.0%	95.8%	100.0%	94.1%
5F	59	0	60	1	98.3%	100.0%	99.2%	100.0%	98.4%
6F	88	0	20	12	88.0%	100.0%	90.0%	100.0%	62.5%
7F	5	0	115	0	100.0%	100.0%	100.0%	100.0%	100.0%
8F	9	1	109	1	90.0%	99.1%	98.3%	90.0%	99.1%
9F	20	0	100	0	100.0%	100.0%	100.0%	100.0%	100.0%
10F	40	0	80	0	100.0%	100.0%	100.0%	100.0%	100.0%
11F	60	0	60	0	100.0%	100.0%	100.0%	100.0%	100.0%
12F	79	0	20	21	79.0%	100.0%	82.5%	100.0%	48.8%
13F	4	0	115	1	80.0%	100.0%	99.2%	100.0%	99.1%
14F	8	0	110	2	80.0%	100.0%	98.3%	100.0%	98.2%
15F	18	0	100	2	90.0%	100.0%	98.3%	100.0%	98.0%
16F	38	0	80	2	95.0%	100.0%	98.3%	100.0%	97.6%
17F	60	0	60	0	100.0%	100.0%	100.0%	100.0%	100.0%
18F	96	13	7	4	96.0%	35.0%	85.8%	88.1%	63.6%
1M	5	0	115	0	100.0%	100.0%	100.0%	100.0%	100.0%
2M	6	0	110	4	60.0%	100.0%	96.7%	100.0%	96.5%
3M	12	0	100	8	60.0%	100.0%	93.3%	100.0%	92.6%
4M	18	0	80	22	45.0%	100.0%	81.7%	100.0%	78.4%
5M	54	0	60	6	90.0%	100.0%	95.0%	100.0%	90.9%
6M	96	0	20	4	96.0%	100.0%	96.7%	100.0%	83.3%
7M	3	0	115	2	60.0%	100.0%	98.3%	100.0%	98.3%
8M	9	0	110	1	90.0%	100.0%	99.2%	100.0%	99.1%
9M	13	0	100	7	65.0%	100.0%	94.2%	100.0%	93.5%
10M	34	0	80	6	85.0%	100.0%	95.0%	100.0%	93.0%
11M	38	0	60	22	63.3%	100.0%	81.7%	100.0%	73.2%
12M	82	0	20	18	82.0%	100.0%	85.0%	100.0%	52.6%
13M	5	0	115	0	100.0%	100.0%	100.0%	100.0%	100.0%
14M	8	0	110	2	80.0%	100.0%	98.3%	100.0%	98.2%
15M	12	0	100	8	60.0%	100.0%	93.3%	100.0%	92.6%
16M	39	0	80	1	97.5%	100.0%	99.2%	100.0%	98.8%
17M	48	0	60	12	80.0%	100.0%	90.0%	100.0%	83.3%
18M	90	0	20	10	90.0%	100.0%	91.7%	100.0%	66.7%
